# Case report: metoclopramide induced acute dystonic reaction in adolescent CYP2D6 poor metabolizers

**DOI:** 10.3389/fphar.2023.1201566

**Published:** 2023-07-11

**Authors:** Franz-Martin Fink, Marta Bognar, Petra Hengl, Markus Paulmichl, Charity Nofziger

**Affiliations:** ^1^ Department of Pediatrics, Regional Hospital, St Johann in Tirol, Austria; ^2^ Department for Personalized Medicine, Privatklinik Maria Hilf, Klagenfurt, Austria; ^3^ PharmGenetix Gmbh, Niederalm, Austria

**Keywords:** CYP2D6, poor metabolizer, metoclopramide, acute dystonia, metoclopramide-induced acute dystonic reactions, pharmacogenetics, pharmacogenomics

## Abstract

Metoclopramide is indicated for the management of gastroesophageal reflux, gastric stasis, nausea, and vomiting. Metoclopramide-induced acute dystonic reactions (MIADRs), along with repetitive involuntary protrusion of the tongue, are well-known phenomena in children and young adults that may appear after the first dose. The drug is primarily metabolized via oxidation by the cytochrome P450 enzyme CYP2D6 and to a lesser extent by CYP3A4 and CYP1A2. A recommendation to decrease metoclopramide dosing in patients with severely limited to no CYP2D6 activity (i.e., poor metabolizers, PMs) is included in the drug label. It is important to note, however, that a requirement or recommendation for pre-emptive testing for CYP2D6 metabolizer status is not included in the drug label. We present two cases of acute dystonia in two non-consanguineous male adolescents: one following metoclopramide and cimetidine administration in a 14-year-old to treat gastroesophageal reflux, and another following metoclopramide and pantoprazole administration in a 17-year-old with acute gastroenteritis. A retrospective pharmacogenetic analysis revealed both patients as CYP2D6 PMs.

## Introduction

Metoclopramide is a dopamine receptor antagonist approved for the management of gastrointestinal distress (nausea, vomiting, *etc.*) with an i) increase in the lower esophageal sphincter pressure, ii) increase in the amplitude of both the esophageal and gastric antrum peristalsis, iii) relaxation of the pyloric sphincter, and iv) increase in the small-bowel transit via its significant modulation of the cholinergic nervous system (Schulze-Delrieu, 1981; Desmond and Watson, 1986). The drug also has a central effect on the chemoreceptor trigger zone and effects on the release of various hormones (Schulze-Delrieu, 1981; Desmond and Watson, 1986). It is rapidly absorbed into the gastrointestinal tract with a bioavailability varying between 35% and 100% depending on the extent of first-pass metabolism, the elimination half-life varies between 3 and 6 hours, and protein binding is low (Desmond and Watson, 1986). Metoclopramide metabolism involves oxidation (by the cytochrome P450 enzymes CYP2D6, CYP3A4, and CYP1A2) and, albeit to a lesser extent, glucuronide and sulfate conjugation by the UDP-glucuronosyltransferase (UGT) and sulfotransferase (SULT) (Argikar et al., 2010; Parkman et al., 2012; Livezey et al., 2014; Ge et al., 2020).

Metoclopramide-induced acute dystonic reactions (MIADRs), along with repetitive involuntary protrusion of the tongue, is a well-known phenomenon in children and young adults that may appear after the first dose, and includes involuntary movements of limbs, facial grimacing, torticollis, oculogyric crisis, bulbar type of speech, trismus, opisthotonus (tetanus-like reactions), and, rarely, stridor and dyspnea possibly due to laryngospasm (Bateman et al., 1989).

It is well known that underlying single nucleotide variants (SNVs) and other structural variations in *CYP2D6* contribute to altered metabolizing capacities for this enzyme in some individuals (Nofziger et al., 2020). As such, oxidative metoclopramide metabolism could be reduced in patients without full CYP2D6 enzyme function. This is supported by the recommendation within the drug’s label that its dose should be reduced in CYP2D6 PMs, other case reports describing metoclopramide-induced adverse side effects in CYP2D6 PMs (van der Padt et al., 2006; Chua et al., 2019), and pharmacokinetic studies of metoclopramide and CYP2D6 (Bae et al., 2020).

Here, we describe additional cases of MIADR in adolescent CYP2D6 PMs.

## Case description

### Case 1

**FIGURE 1:**
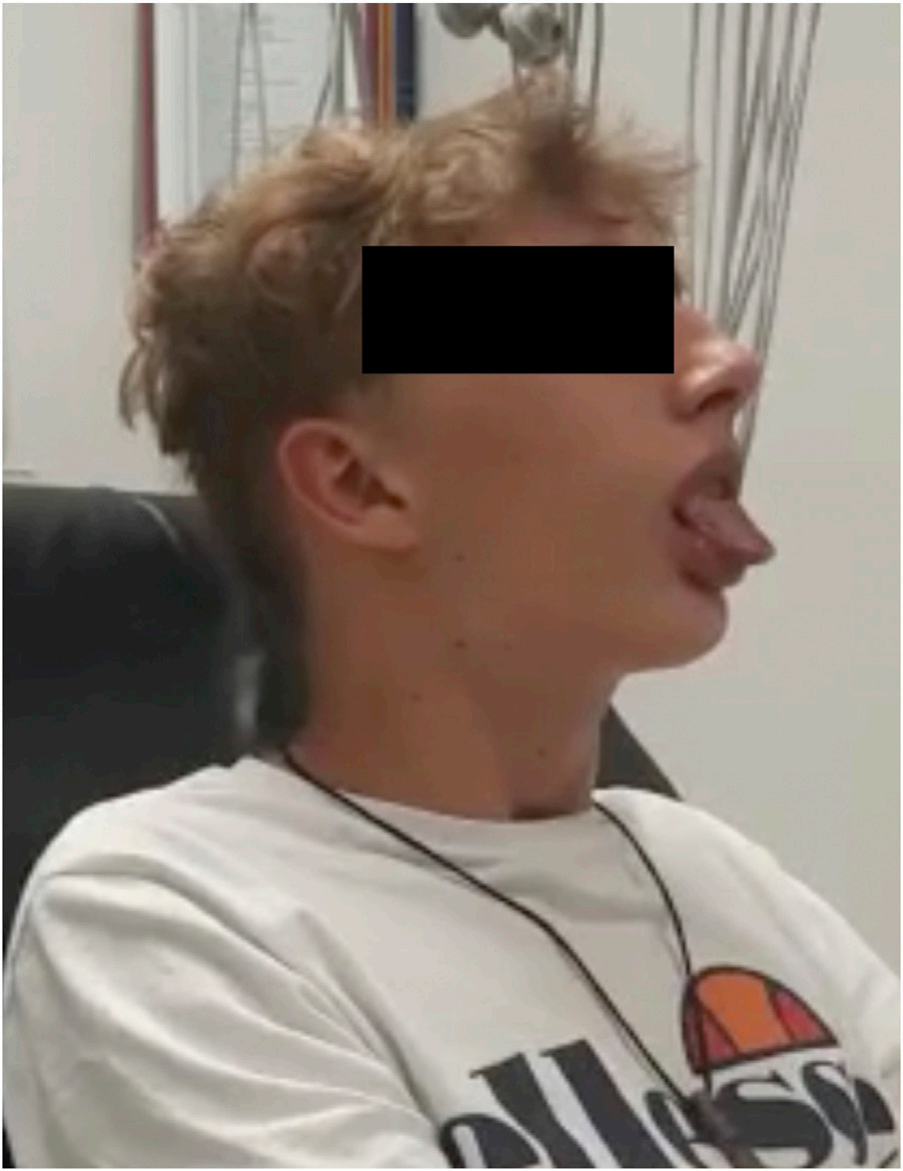
Visual example of facial hypotonia (with open mouth most of the time and repeated involuntary protrusion of the tongue) from Case 1.

A 14-year-old, 62 kg male of Caucasian ancestry was admitted to the hospital with suspected seizures. He presented cooperative and fully conscious but with slurred speech and facial hypotonia with open mouth most of the time and repeated involuntary protrusion of the tongue (Figure 1). In addition, he complained of dystonic muscular spasms in the left side of the neck with intermittent retroflexion of the head. Because of the negation of any drug intake, the differential diagnostic spectrum included neurologic (paroxysmal dystonia) and psychiatric disease. A 3.4 mg oral dose of diazepam terminated the bizarre clinical episode. Then, 12 h later, after a calm night, the patient was well and without any clinical signs of disease. Only now the patient disclosed that 3 days before the hospital admission, he was started on metoclopramide (10 mg twice daily) and cimetidine (400 mg once daily) for gastroesophageal reflux. Exposure to metoclopramide prior to this case was not reported. A retrospective pharmacogenetic analysis was recommended to identify the reason for the suspected seizures and dystonia, and revealed a *CYP2D6* diplotype of *68+*4/*5, which confers a PM phenotype (no residual function of CYP2D6).

### Case 2

A 17-year-old, 63 kg male of Caucasian ancestry with acute gastroenteritis was administered metoclopramide OTC from the pharmacy. Immediately following the first 10 mg dose, the patient vomited. The consulted physician prescribed three doses of 10 mg metoclopramide, which were taken 4 and 5 h apart, respectively, together with 40 mg pantoprazole once daily. After the second dose of metoclopramide, mild torticollis was recognized by the patient’s father. In the evening of the same day, 2 hours after the third metoclopramide dose, the patient presented at an outpatient emergency center with a stiff neck and cervical muscle spasms, abnormal eye movements, and bilateral blepharospasm. Metoclopramide-induced acute dystonia with facial grimacing, torticollis, muscle spasms, and oculogyric crisis was diagnosed. A 3.4 mg oral dose of diazepam terminated the extrapyramidal movement disorder. Exposure to metoclopramide prior to this case was not reported. A retrospective pharmacogenetic analysis revealed that this patient was also a CYP2D6 PM, with a diplotype of *4/*68+*4.

## Discussion

MIADRs are well-known adverse drug reactions (ADRs) in children and young adults. The respective summary of product characteristics (SmPCs) in different regulatory environments (United States of America, EU, *etc.*) mentions the important role of metoclopramide metabolism for the occurrence of ADRs. Metoclopramide is predominantly metabolized by the liver enzyme CYP2D6 (Livezey et al., 2014). As such, oxidative metoclopramide metabolism is expected to be reduced in patients without CYP2D6 enzyme function (i.e., PMs), which is underscored by the recommendation from the FDA that metoclopramide dosing should be reduced in CYP2D6 PMs, and should not be co-administered with strong CYP2D6 inhibitors (U.S. Food and Drug Administration, 2017). In 2014, the European Medicines Agency (EMA) issued a press release restricting the use of metoclopramide to short-term only (up to 5 days) due to “…well-known risks of neurological effects such as short-term extrapyramidal disorders … ” and recommended that the drug should not be used in children below 1 year of age and that in older children it should be used only as a second choice treatment for chemotherapy-induced emesis (European Medicines Agency, 2013). Interestingly, the press release made no mention of dosing guidance with respect to the CYP2D6 metabolizing capacity.

It may also be important to note that the role of UGTs and SULTs, both important for the metabolism of various xenobiotics (i.e., paracetamol, anticancer drugs, etc.), in metoclopramide-related toxicities was highlighted as the main detoxifying metabolic pathways of the drug, followed by renal elimination of the more soluble metabolites (including 20% of the unchanged drug) (Ge et al., 2020). However, recent data, including that presented here from patients with no function of CYP2D6 (i.e., CYP2D6 PMs), underline the importance of CYP2D6 function for the development of metoclopramide-induced ADRs and is further substantiated by a recent case report describing metoclopramide-associated dystonia in a patient with an intermediate CYP2D6-metabolizing phenotype (Wong and Fogel, 2021), as well as a separate study showing increased metoclopramide plasma concentrations in CYP2D6 PMs and IMs compared to normal metabolizers (Bae et al., 2020). The observed frequency of CYP2D6 PMs in Europeans is as high as 8.45%, and the predicted frequency of CYP2D6 IMs in Europeans is as high as 35.3% (Gaedigk et al., 2017). However, 2 years after this publication, the method for calculating activity scores for CYP2D6 IMs changed, and the activity value of the relatively common CYP2D6*10 allele was lowered from 0.5 to 0.25 (Caudle et al., 2020). Therefore, the frequency of CYP2D6 IMs in Europeans reported by Gaedigk et al. (2017) may be underestimated.

The PM status of CYP2D6 in the aforementioned patients was likely the primary driver of the observed metoclopramide-induced acute dystonic episodes. Co-administration of cytochrome P450 inhibitors may transform NMs into IMs or PMs (phenoconversion) (Klomp et al., 2020). In both patients, the co-administration of cimetidine (inhibitor of CYP2D6, CYP3A4, and CYP1A2) and pantoprazole (inhibitor of CYP3A4), in case 1 and 2, respectively, may have further augmented the MIADR by decreasing the ability of the patients to metabolize metoclopramide (Martinez et al., 1999; Li et al., 2004).

## Conclusion

With such a high percentage of the European population expected to have significant deficiencies in CYP2D6 function, the risk for developing ADRs after taking metoclopramide is notable, especially in the vulnerable population of children. Therefore, it seems warranted that the European regulatory environment adopts respective specific information regarding the importance of CYP2D6 function for the risk of development of metoclopramide-associated ADRs.

A pharmacogenetic test for CYP2D6 phenotype prediction enables the physician to adopt the prescription of drugs which are likely not well-tolerated. Based on this genetic information, dose reduction or the choice of an alternative drug might have prevented the ADRs described in this case report. In addition, whenever two or more drugs are administered simultaneously, the risk of phenoconversion should be considered.

## Data Availability

The raw data supporting the conclusions of this article will be made available by the authors, without undue reservation.
